# Salient omissions—pupil dilation in response to unexpected omissions of sound and touch

**DOI:** 10.3389/fpsyt.2023.1143931

**Published:** 2023-03-22

**Authors:** Tjerk T. Dercksen, Andreas Widmann, Nicole Wetzel

**Affiliations:** ^1^Research Group Neurocognitive Development, Leibniz Institute for Neurobiology, Magdeburg, Germany; ^2^Center for Behavioral Brain Sciences, Magdeburg, Germany; ^3^Wilhelm Wundt Institute for Psychology, Leipzig University, Leipzig, Germany; ^4^University of Applied Sciences Magdeburg-Stendal, Stendal, Germany

**Keywords:** pupillometry, omission, surprise, prediction error, predictive coding, auditory, somatosensory

## Abstract

**Introduction:**

Recent theories describe perception as an inferential process based on internal predictive models adjusted by means of prediction violations (prediction error). To study and demonstrate predictive processing in the brain the use of unexpected stimulus omissions has been suggested as a promising approach as the evoked brain responses are uncontaminated by responses to stimuli. Here, we aimed to investigate the pupil’s response to unexpected stimulus omissions in order to better understand surprise and orienting of attention resulting from prediction violation. So far only few studies have used omission in pupillometry research and results have been inconsistent.

**Methods:**

This study adapted an EEG paradigm that has been shown to elicit omission responses in auditory and somatosensory modalities. Healthy adults pressed a button at their own pace, which resulted in the presentation of sounds or tactile stimuli in either 88%, 50% or 0% (motor-control) of cases. Pupil size was recorded continuously and averaged to analyze the pupil dilation response associated with each condition.

**Results:**

Results revealed that omission responses were observed in both modalities in the 88%-condition compared to motor-control. Similar pupil omission responses were observed between modalities, suggesting modality-unspecific activation of the underlying brain circuits.

**Discussion:**

In combination with previous omission studies using EEG, the findings demonstrate predictive models in brain processing and point to the involvement of subcortical structures in the omission response. Our pupillometry approach is especially suitable to study sensory prediction in vulnerable populations within the psychiatric field.

## 1. Introduction

Unexpected events, such as a loud noise (1), a sudden plane engine failure (2), or a sports match that takes an unexpected turn (3), are typically followed by a dilation of the pupil. This pupil dilation response (PDR) is thought to reflect a physiological reaction to surprise and has been observed in a wide range of contexts (4–9). Surprise responses have played a principal role in traditional and modern theories of brain function, for instance as a reflection of the orienting response (10, 11) or as a consequence of prediction error in the predictive coding framework (12). The “oddball” paradigm is commonly used to elicit surprise in controlled settings, in which a repeated standard stimulus is occasionally interrupted by a rare and unexpected deviant stimulus. Numerous studies have shown an increased PDR in deviant compared to standard stimuli (13–15) which is associated with increased activity in the locus coeruleus norepinephrine (LC-NE) system and the superior colliculus (16–21).

One challenge in using pupil dilation as a measure of surprise in response to deviant stimuli is that these stimuli may also affect pupil size through various other mechanisms. For example, a novel sound in a series of standard sounds may require additional cognitive resources for processing, or an unfamiliar environmental sound may elicit encoding of new memories. These and other stimulus-related factors can impact pupil dilation (6, 22), but may not necessarily be related to surprise. As a result, the conflation of stimulus- and surprise-related factors on the low-dimensional measure of pupil dilation can make it difficult to draw conclusions about surprise alone when analyzing the PDR.

An innovative approach to studying surprise is through the use of stimulus omissions, where expected standard stimuli are occasionally replaced by an unexpected stimulus absence to elicit surprise. The surprise response to omission can be explained in terms of predictive coding, a neurocognitive theory that posits that the brain uses prior knowledge to continually generate predictions about incoming sensory information. When these predictions are incorrect compared to actual sensory input, this results in a prediction error or surprise response that is used to update and refine the brain’s internal models, allowing for more accurate predictions in the future (12, 23, 24). Omission studies use these principles to construct experiments in which a prediction of a stimulus is built and then the stimulus is unexpectedly omitted, resulting in a discrepancy between prediction and input and therefore a surprise response. This approach avoids confounding factors related to the stimulus itself, allowing for a more precise analysis of the effects of surprise on neural processing or behavior.

While theoretically appealing, the use of stimulus omissions has produced somewhat inconsistent results in previous pupillometry research. Cooper et al. (25) observed only rare pupil responses to auditory omissions in paralyzed cats [but note that the relationship between subcortical activity and pupil responses differs substantially between species, e.g., (18, 26)]. Stemmerding et al. (27) observed responses to the omission of fear stimuli in the skin conductance response but not in the PDR. Damsma and Van Rijn (28) observed an amplified PDR only for omission of the most salient sound on the first beat of a drum sequence but not for the second beat or hi-hat sounds. Zhang et al. (29), on the other hand, show convincing pupillary omission responses when coupling visual and auditory stimuli where occasionally the visual stimulus was omitted. This suggests that pupil responses to omissions can be observed under the right conditions. However, it is not yet clear what these conditions may be. In contrast, studies using EEG have consistently demonstrated robust activations in response to omissions over the past years (30–37). Moreover, the omission P3 (oP3) component that is elicited in response to omissions in the EEG seems to resemble the novelty or surprise evoked P3a. This component is typically also associated with pupil dilation in response to unexpected stimuli and presumably reflects processes related to attention orienting (38, 39). An important difference between pupillometry and EEG omission studies is the condition to which the omission is compared. In pupillometry studies, unexpected omissions have typically been compared to standard stimuli (27–29), while in EEG studies, unexpected omissions are compared to expected omissions. This reintroduces stimulus-specific confounds to pupillometry studies and may have contributed to inconsistent results in the past. Furthermore, recent omission studies using EEG typically use a time-locking cue, such as the action of a button press, to indicate the exact moment when a stimulus should have occurred. Actions have repeatedly shown to trigger strong predictions of associated effects [see (40) for a review], but stimuli in different modalities seem to be able to serve the same purpose (36). Using such time-locking cues likely avoids temporal shifts in the omission response, which may have led to null results in previous studies (41).

Pupil dilation studies have predominantly focused on the visual and auditory modalities, with comparatively few examining the somatosensory modality (42). Similar to other modalities, the pupil responds to tactile stimuli [e.g., (43)]. While some pupillometry studies have investigated surprise responses to tactile stimuli (11, 44), these have largely been in the context of pain research and little is known about more general tactile surprise processes. To the best of the authors’ knowledge, surprise responses have not yet been directly compared across the auditory and somatosensory modalities using pupillometry. This is particularly interesting given that Dercksen et al. (30) recently identified similar omission components in the EEG using tactile stimuli as those previously recorded using auditory stimuli.

The current study utilizes a tried-and-tested paradigm adapted from EEG studies to examine omission responses in the auditory and somatosensory modalities using pupillometry. This paradigm has been proven to consistently elicit EEG responses to omission in both modalities (30, 34). The study presents three conditions, in which participants are asked to repeatedly press a button. In one condition, the stimulus is coupled with the button press most of the time (88%-condition). In the other two conditions, the coupling is either unpredictable (50%-condition) or absent (motor-control condition). Omission responses are expected to occur only in the 88%-condition, as there is a prediction of a stimulus, resulting in surprise when it is omitted. The other two conditions serve as proper comparisons by examining equivalent actions (button presses without a stimulus) where only the surprise associated with the omission is varied.

## 2. Materials and methods

### 2.1. Participants

A total of 40 participants took part in the experiment (29 female; age range = 18–35; mean age = 24; SD = 4.4 years). All participants were right-handed, as measured by a German version of the Oldfield Scale (45). All participants reported normal hearing and touch and were compensated either financially or in the form of course credit points. Participants gave written consent prior to the experiment. The project was approved by the local ethics committee.

### 2.2. Stimuli and apparatus

#### 2.2.1. Sound stimuli

A total of 48 different common environmental sounds (e.g., dog, car-horn, trumpet) rated as identifiable by an independent sample of participants [in 200 ms form, see (46)] were used as sound stimuli. Sounds were presented binaurally for 200 ms using Sennheiser HD-25 headphones and were tapered-cosine windowed (10 ms rise- and 10 ms fall-time) and root mean square (RMS) matched. Loudness was set at 70.4 dB SPL for all participants [identical to (31)]. A new sound was presented for each auditory experimental block, where all 48 sounds were balanced across participants.

#### 2.2.2. Tactile stimuli

Presentation of tactile stimuli was performed using pulses of pressurized air (3 bar) that inflated a membrane, which was controlled using a somatosensory stimulus generator (University of Münster, Germany) that was placed outside the chamber. Tactile stimulus duration was approximately 30 ms. Two membranes were placed on the left middle and index fingers at the volar aspect of the distal phalanx [identical to (30)]. The tactile stimulus always consisted of simultaneous stimulation of both fingers. Because of the travel time of the air pulse, there was a slight time delay between button press and inflation of the membrane (onset of the tactile stimulus) of approximately 40 ms.

#### 2.2.3. Apparatus

Participants were seated in an electrically shielded and acoustically attenuated chamber, where a constant luminance of 48.9 lx (measured with MAVOLUX 5032B USB, GOSSEN Foto- and Lichtmesstechnik GmbH, Nürnberg, Germany) was maintained. Pupil diameter of both eyes was recorded with an infrared EyeLink Portable Duo eye-tracker (SR Research Ltd., Mississauga, Ontario, Canada). The eye tracker was set up in remote mode at a sampling rate of 500 Hz. The experiment was programmed using Psychtoolbox (version 3.0.15; 47) and ran on a Linux-based system using GNU Octave (version 4.0.0). A white fixation cross was presented using a VIEWPixx/EEG Display (Resolution 1920(H) x 1,080(V)—23.6-inch display size). The fixation cross was presented in the middle of a grey screen (illuminance: 13.1 cd/m^2^), at about 60 cm from the participants’ eyes (0.67°× 0.67° visual angle). To trigger the stimuli (or omissions), a custom-built button was used in order to ensure a completely silent button press. The button used an infrared photoelectric mechanism and was additionally padded with sound absorbing material. To ensure that no residual sound (e.g., contact of the skin of the fingertip with the button surface) was correlated with the button press and membrane inflation, participants wore the above mentioned Sennheiser HD-25 headphones throughout the experiment (also when no sounds were presented).

### 2.3. Procedure

**Figure 1:**
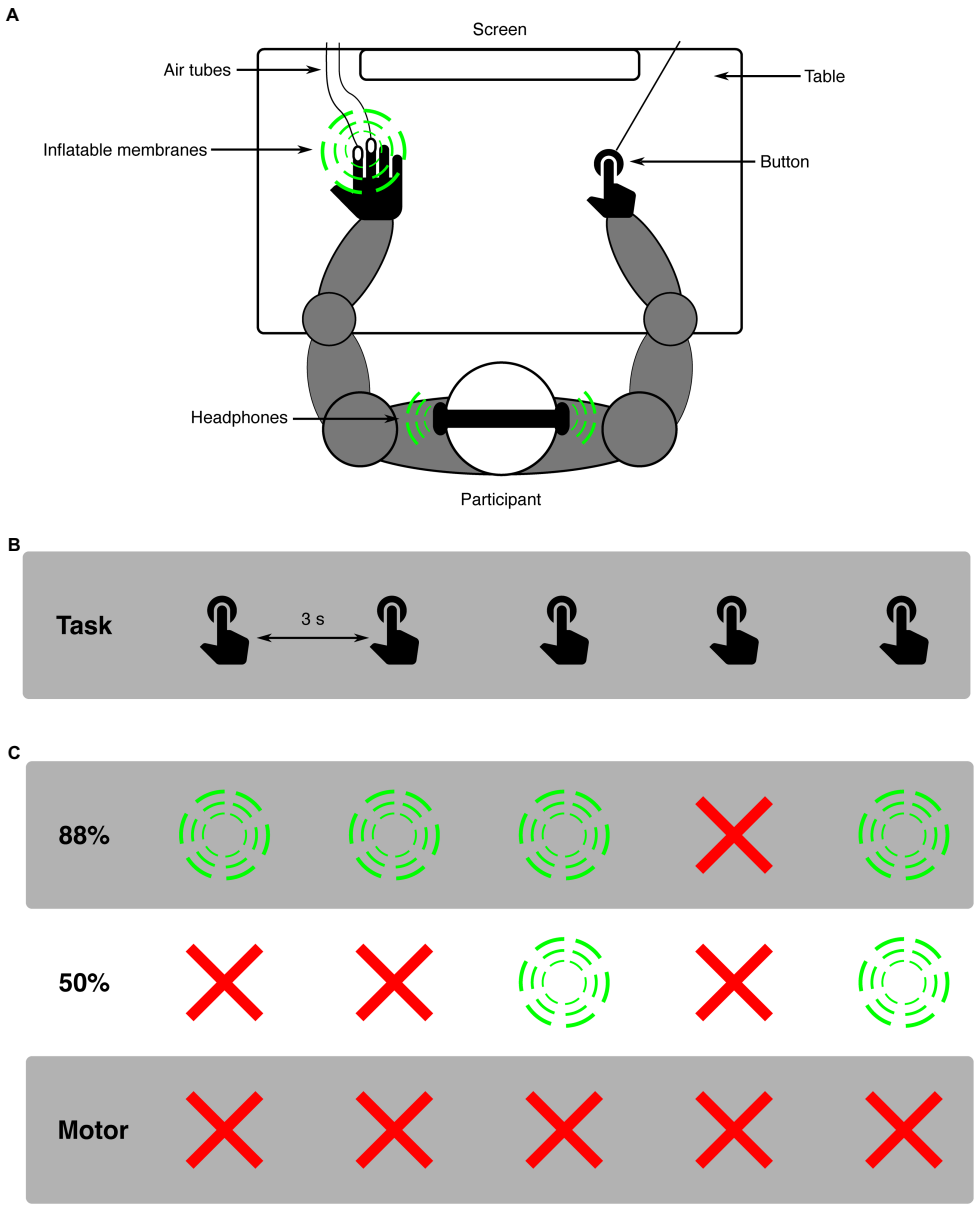
Schematic representation of the experimental design. **(A)** shows the experimental set-up: a participant sat in front of a screen with both arms on a table. With the right hand a button was pressed, possibly resulting in a stimulus (indicated with green circles). The same stimulus modality was always presented in one experimental block, either auditory or somatosensory. Auditory stimuli were presented using headphones. Somatosensory stimuli were applied by a puff of air traveling through air tubes and inflating a membrane on the left middle- and index-finger*s*. **(B)** depicts the task over time, where participants aimed to press a button every 3,000 ms. **(C)** shows examples of the effects of the button presses for all three conditions (green circles are stimulus presentations, red crosses are omissions). In the 88%-condition, there was an 88% chance of a button press resulting in a stimulus.

Participants were seated approximately 60 cm from a screen, having their right index finger on a button, their left hand on a table (with membranes attached for applying tactile stimuli), and wearing headphones (see Figure 1 for experimental set-up). In all conditions, participants were asked to press a button every 3,000 ms while looking at a fixation cross that was presented on a screen. For both modalities (auditory and somatosensory), two distinct conditions (88%-condition and 50%-condition) were presented. An experimental block always presented only one modality and condition (e.g., auditory 88%-condition). In the 88%-condition, 88% of button presses resulted in a stimulus, while the remaining 12% of button presses resulted in omissions. Omissions were randomly placed within the block to ensure unpredictability, with the only restricting conditions that the first two button presses of every block and the two button presses following an omission always resulted in a stimulus. This was done to avoid any persisting attention- or deviance-related effects in responses to standard stimuli [similar to, e.g., (30)]. In the 50%-condition, 50% of button presses resulted in a stimulus and the other 50% in omissions. For this condition no restrictions were applied. Additional to the 88%- and 50%-conditions, a motor-control condition was applied to analyze the effect of pressing the button on the pupil. In this condition only the button was pressed, never resulting in a stimulus (i.e., 100% omission). Before the experiment, two short training blocks (15 trials each block) were completed where participants trained to press the button every 3 s. In the first training block, visual feedback on the screen was given after each button press about the time between the current and previous button presses. In the second training block, the participant practiced to keep the correct time between button presses without visual feedback. If participants did not report confidence in their ability to press in the appropriate rhythm, training blocks were repeated to provide additional practice in keeping the time between button presses around 3 s. After training, 12 experimental blocks were presented. Modalities were always presented separately, i.e., first all blocks for one modality were presented, followed by all blocks presenting the other modality, which was balanced across participants. Within a modality, five 88%-condition blocks, one 50%-condition block, and one motor-control condition block were presented. The five 88%-condition blocks were always presented in direct succession. The modality would start with either a 88%-condition or 50%-condition block (balanced across participants), which was followed by a motor-control block, which was then followed by the remaining condition (either 88%- or 50%-condition). The separation between 88%- and 50%-conditions by a motor-control block was implemented to minimize possible carry-over effects between conditions within a modality. Blocks in the 88%-condition consisted of 66 trials, presenting 58 stimulus (sound or tactile) and 8 omission trials. Blocks in the 50%-condition consisted of 80 trials, presenting 40 stimulus (sound or tactile) and 40 omission trials. Blocks in the motor-control condition consisted of 40 trials. The slight increase in the number of trials for the 50%-condition block compared to the 88%-condition block was chosen so that all required trials in the 50%-condition could be presented in a single block. The decrease in the number of trials for the motor-control condition block compared to the 88%-condition block was chosen so that all required trials in the motor-control condition could be presented in 2 blocks that were spread out in the experiment. A total of 768 trials were presented: 528 for 88%-condition (64 omissions), 160 for 50%-condition (80 omissions) and 80 for motor-control (80 omissions). Participants were instructed about the details of the upcoming condition (whether auditory or somatosensory and 88, 50% or motor condition was presented) before the respective block started. Total experiment time was around 70 min including breaks.

### 2.4. Pupil data preprocessing

Pupil diameter measurements were converted to mm as suggested by Steinhauer et al. (48). Eye saccade and blink information were provided by the eye tracker. Partial blinks were detected during post-processing from the smoothed velocity times series by an additional custom function. This function detected pupil diameter changes exceeding 20 mm/s including a 50 ms pre-blink and a 100 ms post-blink interval [as suggested by Merritt et al. (49)]. Data from both eyes was averaged using the dynamic offset algorithm (50). Blinks and other intervals with signal loss longer than 1 s were discarded from the data, while shorter intervals were interpolated (using the MATLAB 1-D interpolation function with shape-preserving piecewise cubic interpolation). Data was segmented in 2 s epochs around the triggers of interest including a 0.2 s pre-stimulus baseline period (total time window: −0.2 s to 1.8 s). Epochs were baseline corrected by subtracting the mean amplitude from the baseline period [− 0.2 s to 0 s, similar to (13, 51)]. The first 2 trials for all blocks and the 2 trials immediately following an omission in the 88%-condition were removed from further analysis. Individual mean PDRs were computed per participant and condition. For statistical testing, mean PDRs were computed from a time window around the peak between 0.6 s and 0.8 s after button press, which is similar to time windows where other omission studies have found effects (28, 29, 52).

### 2.5. Statistics and data analyses

#### 2.5.1. Statistics

Statistical analyses were performed on the PDR data using a Bayesian approach. Additionally, we report frequentist statistics for informational purposes. This way, readers familiar with Bayesian statistics can benefit from its advantages (53, 54) while still keeping results interpretable for readers more familiar with frequentist statistics. Statistical testing was done using JASP (version 0.16.4, 55). For Bayesian *t*-tests, either a one sample test (to compare with motor activity) or two-tailed paired test (to compare between modalities) were used where the null hypothesis corresponded to a standardized effect size δ = 0, while the alternative hypothesis was defined as a Cauchy prior distribution centered around 0 with a scaling factor of *r* = 0.707 (the default “medium” effect size prior scaling). For Bayesian repeated-measures ANOVA [rANOVA; see (56) for more information on Bayesian ANOVA], the JASP default fixed effects priors, random effects priors and covariates priors were used, defined as, respectively, *r* = 0.5, *r* = 1 and *r* = 0.354. Bayesian rANOVA tested all alternative models (main effects and interactions) against the null model, which included subjects and random slopes. The *BF*_inclusion_ factor across matched models was calculated for all variables to determine the evidence provided by the data for an effect if comparing all matched models including vs. excluding the effect. Bayes Factor (*BF*_10_) was calculated using 10.000 sample repetitions (the JASP default) and was interpreted following Lee & Wagenmakers (57), who give the labels anecdotal (0.33–3), moderate (3–10 or 0.33–0.1), strong (10–30 or 0.1–0.033), and very strong (>30 or < 0.033) for specific ranges of the Bayes Factor. We replaced the label “anecdotal” with “weak,” and “very strong” with “decisive” to aid interpretation. The direction of the effect was only reported if the alternative model was preferred over the null model by the data (i.e., BF_10_ > 1). For frequentist *t*-tests and rANOVA effect size was reported using Cohens *d* and the generalized η^2^ [η_G_^2^; (58)] respectively.

#### 2.5.2. Planned data analysis

An initial data analysis was planned *a priori* and aimed to replicate the analysis strategy from preceding EEG studies (30–32, 34, 35). These studies in a first step typically compared omissions in the 88%-condition to the motor-control condition as well as omissions in the 50%-condition to the motor-control condition to confirm whether omission responses were elicited in the respective conditions. Presence of an effect in one and absence in another condition does not necessarily indicate a significant difference of the effect between conditions [see (59) for discussion], which is why in a second step typically omission responses were directly compared between the 88%-condition and the 50%-condition to confirm that larger omission responses were elicited in the 88% condition. In comparison to the preceding EEG studies in the present study, the additional factor modality (auditory and somatosensory) was introduced. In the first step we therefore tested the omission PDR amplitudes averaged over modalities against the PDR amplitudes in the motor-control condition separately for each probability condition (88% and 50%) and next the omission PDR amplitudes in the auditory against the somatosensory condition separately for each probability condition.^1^ In the second step, we then tested differences between 88%-condition and 50%-condition using a 2 × 2 rANOVA including the factors probability condition (88% and 50%) and modality (auditory, somatosensory).

#### 2.5.3. Post-hoc data analysis

A second, post-hoc data analysis of omission results was decided upon *a posteriori*. We could not help but notice block-specific effects in the 88% and motor-control conditions, showing a substantial attenuation of the PDR after the first block. This observation implies a confounded comparison between omission conditions in the planned data analysis, as the 88%-condition was presented in five consecutive blocks whereas the 50%-condition was presented in a single block. Therefore, we performed another, post-hoc data analysis that took habituation effects into account. First, we tested PDR amplitudes in the 88% and motor-control condition observed in the first block against the PDR amplitudes in the following blocks (second block in motor-control and second to fifth block in 88% condition) to confirm the habituation effects. Next, we replicated all analyses of the planned data analysis (see above) on the data observed in the first block per condition only.

## 3. Results

### 3.1. Behavior

Participants were generally able to keep a stable pace between button presses throughout the experiment, where the aim was to keep inter-press interval around 3 s. Group average for motor-control condition was 2.77 s (SD = 0.41 s), for auditory 88%-condition 2.75 s (SD = 0.38 s), for auditory 50%-condition 2.85 s (SD = 0.44 s), for somatosensory 88%-condition 2.80 s (SD = 0.43 s) and for somatosensory 50%-condition 2.82 s (SD = 0.43 s).

### 3.2. Pupil dilation responses

#### 3.2.1. Planned data analysis

##### 3.2.1.1. 88%-condition versus motor-control

**Figure 2:**
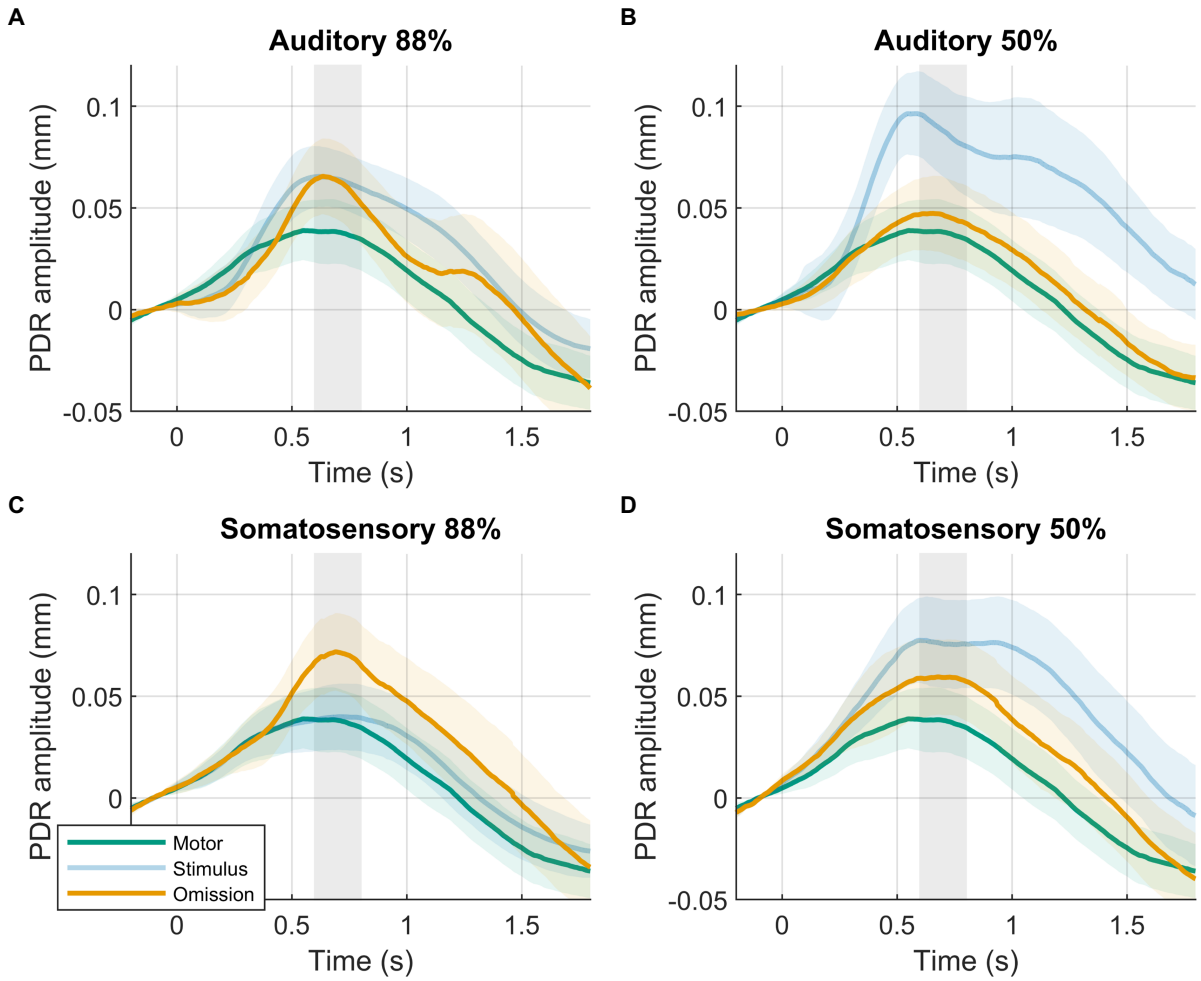
Results of planned analysis. PDRs +95% confidence intervals of motor-control (green), stimulus (blue, transparent) and omission (orange) trials. **(A)** Auditory 88%-condition. **(B)** auditory 50%-condition. **(C)** Somatosensory 88%-condition. **(D)** Somatosensory 50%-condition. Time-window for statistical analysis shown in grey.

In line with our hypothesis, we observed larger PDR amplitudes in response to omissions in the 88%-condition compared to the motor-control condition (Figure 2). The data provided decisive evidence for a condition effect (88%-condition vs. motor-control: BF_10_ = 63, *d* = 0.607, *t*(39) = 3.839, *p* < 0.001). PDR amplitudes in response to omissions in the 88%-condition were similar between modalities. The data provided moderate evidence against an effect of modality (88%-condition, auditory vs. somatosensory: BF_10_ = 0.241, *d* = 0.136, *t*(39) = 0.860, *p* = 0.395).

##### 3.2.1.2. 50%-condition versus motor-control

Although we hypothesized similar PDR amplitudes between omissions in the 50%-condition compared to the motor-control condition, results were not clear, showing slightly larger PDR amplitudes in response to omissions in the 50%-condition compared to the motor-control condition. The data provided inconclusive evidence for a condition effect (50%-condition vs. motor-control: BF_10_ = 1.194, *d* = 0.330, *t*(39) = 2.086, *p* = 0.044). PDR amplitudes in response to omissions in the 50%-condition were similar between modalities. The data provided weak evidence against an effect of modality (50%-condition, auditory vs. somatosensory: BF_10_ = 0.411, *d* = 0.218, *t*(39) = 1.382, *p* = 0.175).

##### 3.2.1.3. 88%-condition versus 50%-condition

Contrary to our hypothesis, we observed similar PDR amplitudes in response to omissions in the 88%-condition compared to omissions in the 50%-condition. PDR amplitudes were similar between modalities. The probability condition (88%-condition vs. 50%-condition) by modality (auditory vs. somatosensory) rANOVA favored the null model. The frequentist rANOVA showed no effects for probability condition (*F*_(1,39)_ = 3.218, *p* = 0.081, *η*_G_^2^ = 0.013), modality (*F*_(1,39)_ = 1.790, *p* = 0.189, *η*_G_^2^ = 0.009), or the probability condition by modality interaction (*F*_(1,39)_ = 0.246, *p* = 0.623, *η*_G_^2^ = 0.000).

#### 3.2.2. Post-hoc data analysis

##### 3.2.2.1. 88%-condition 1^st^ block versus 88%-condition 2+ blocks

**Figure 3:**
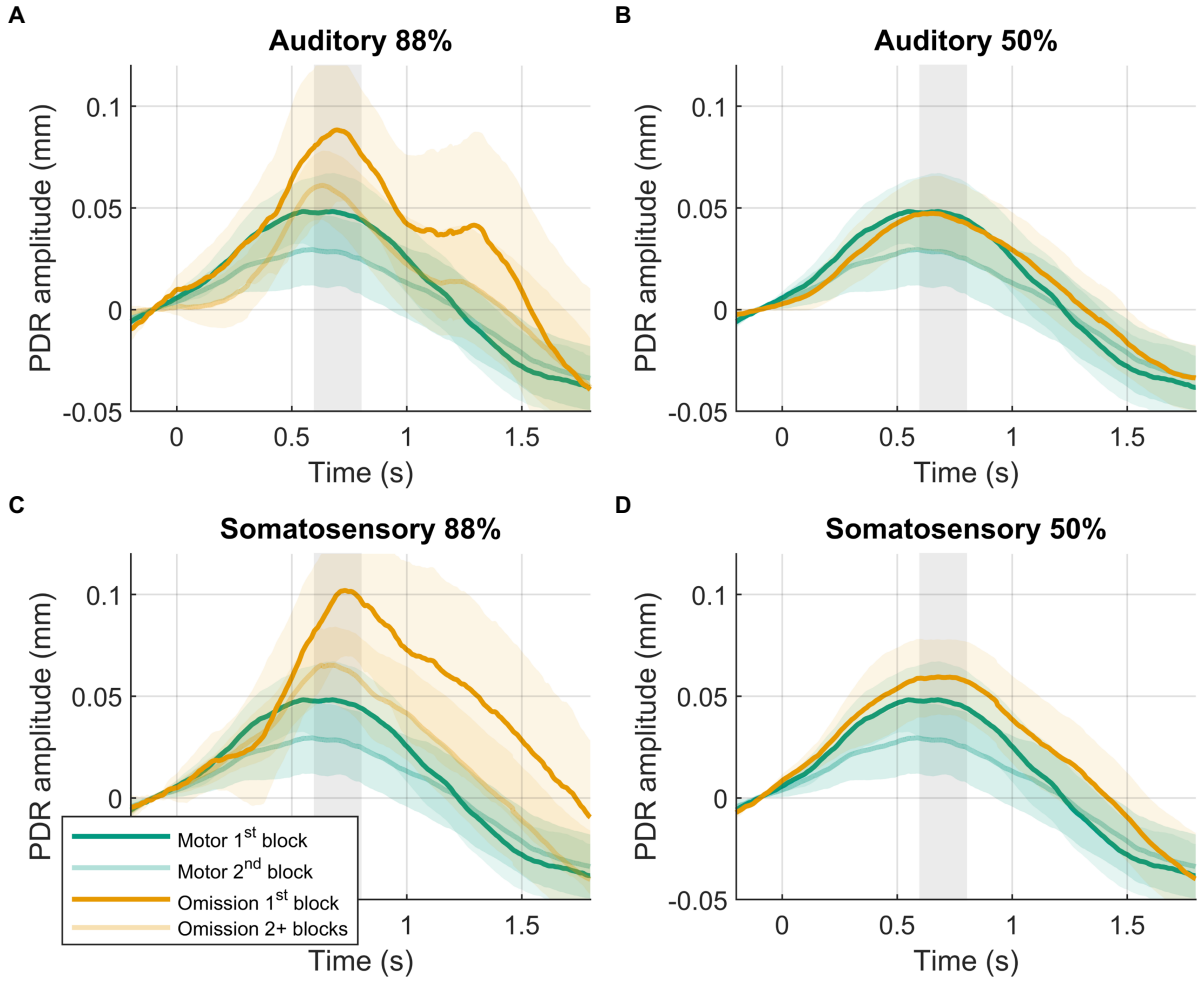
Results of post-hoc analysis. PDRs +95% confidence intervals of motor-control (green) and omission (orange) trials. **(A)** Auditory 88%-condition. **(B)** auditory 50%-condition. **(C)** Somatosensory 88%-condition. **(D)** Somatosensory 50%-condition. PDRs of first block are shown in opaque to show results of interest, following blocks are transparent. Time-window for statistical analysis shown in grey.

Larger PDR amplitudes in response to omissions were observed in the first block of the 88%-condition compared to later blocks in the 88%-condition (Figure 3). PDR amplitudes were similar between modalities. The block (88%-condition 1^st^ block vs. 88%-condition 2+ blocks) by modality (auditory vs. somatosensory) rANOVA favored the model including block, providing moderate evidence (BF_10_ = 5.452). The frequentist rANOVA showed a significant effect for block (*F*_(1,39)_ = 9.091, *p* = 0.005, *η*_G_^2^ = 0.032), no effect for modality (*F*_(1,39)_ = 0.433, *p* = 0.514, *η*_G_^2^ = 0.003), and no modality by block interaction effect (*F*_(1,39)_ = 0.035, *p* = 0.853, *η*_G_^2^ = 0.000).

##### 3.2.2.2. Motor-control 1^st^ block versus motor-control 2^nd^ block

Slightly larger PDR amplitudes were observed in the first block of the motor-control condition compared to the second block of the motor-control condition. The data provided weak evidence for a block effect (BF_10_ = 1.744, *d* = 0.362, *t*(39) = 2.291, *p* = 0.027).

##### 3.2.2.3. 88%-condition 1^st^ block versus motor-control 1^st^ block

In line with earlier hypotheses, we still observed larger PDR amplitudes in response to omissions in the first block of the 88%-condition compared with the first block of the motor-control condition. The data provided decisive evidence for a condition effect (BF_10_ = 135, *d* = 0.651, *t*(39) = 4.120, *p* < 0.001). PDR amplitudes in response to omissions in the first block of the 88%-condition were similar between modalities. The data provided moderate evidence against an effect of modality (BF_10_ = 0.189, *d* = 0.074, *t*(39) = 0.469, *p* = 0.641).

##### 3.2.2.4. 50%-condition versus motor-control 1^st^ block

In line with earlier hypotheses, we now observed similar PDR amplitudes in response to omissions in the 50%-condition compared to the motor-control condition. The data provided moderate evidence against an effect of condition (BF_10_ = 0.219, *d* = 0.116, *t*(39) = 0.731, *p* = 0.469). For differences between modalities, see section 3.2.1: 50% omission versus motor control.

##### 3.2.2.5. 88%-condition 1^st^ block versus 50%-condition

In line with earlier hypotheses, we now observed larger PDR amplitudes in response to omissions in the first block of the 88%-condition compared to omissions in the 50%-condition. PDR amplitudes were similar between modalities. The probability condition (88%-condition 1^st^ block vs. 50%-condition) by modality (auditory vs. somatosensory) rANOVA favored the model including probability condition, providing strong evidence for a larger PDR in the first block of the 88%-condition compared to the 50%-condition (BF_10_ = 12.741). Inclusion Bayes Factor provided strong evidence in favor of including probability condition (BF_inclusion_ = 12.684) but moderate evidence against including modality (BF_inclusion_ = 0.326) and the modality by probability condition interaction (BF_inclusion_ = 0.223). The frequentist rANOVA showed a significant effect for probability condition (*F*_(1,39)_ = 13.534, *p* < 0.001, η_G_^2^ = 0.049), no effect for modality (*F*_(1,39)_ = 0.735, *p* = 0.397, *η*_G_^2^ = 0.005), and no modality by probability condition interaction effect (*F*_(1,39)_ = 0.006, *p* = 0.937, *η*_G_^2^ = 0.000).

## 4. Discussion

The current study aimed to detect a phasic dilation of the pupil in response to unexpected omissions in the auditory and somatosensory modalities. We manipulated the probability of omissions (88%-condition, 50%-condition, motor-control condition), with the 88%-condition being the only one in which the omission was expected to be surprising. Omission responses were observed in the pupil for both modalities when comparing the 88%-condition with the motor-control condition, showing similar omission PDRs across modalities. In contrast, no omission effects were observed when comparing omissions in the 88%-condition with omissions in the 50%-condition. Further analysis revealed that the amount of exposure to a condition (number of experimental blocks) can impact the amplitude of the omission response, hindering detection of the response in the average over all blocks. When the number of presented blocks was matched between conditions, a clear pupil dilation could be observed in the 88%-condition compared with the 50%-condition. In the following we will discuss these results one by one.

### 4.1. Omission responses in 88%-condition compared to motor-control condition

Similar to EEG studies, the current study used a motor-control condition to determine the elicitation of an omission response. In the motor-control condition, none of the button presses result in a stimulus, making the omissions completely predictable. The comparison of omissions in the 88%-condition with the motor-control condition therefore entails a comparison of two identical events (a button press without a stimulus) where only the associated surprise is manipulated. Controlling for motor activity is important even in pupillometry, as actions are known to be accompanied by a dilation of the pupil (60, 61). Also in the current study, a substantial PDR is visible when the button is pressed (Figure 2). The PDR in response to omission in the current study can therefore be defined as the sum of button-press related activity plus a possible surprise response. This can be interpreted as the consequence of high-level executive functions on the one hand and an intermediate-level orienting response on the other in the recent framework proposed by Strauch et al. (8): button-press related pupil dilation likely reflects a combination of task-related, high-level executive attention processes (e.g., temporal attention, pace keeping, decision-making), while the surprise response likely reflects orienting of attention in response to the salient event [presumably similar to the oP3 observed in EEG omission studies, see (30, 32)]. The surprise response was observed in the 88%-condition compared to motor-control for both auditory and somatosensory stimuli (Figures 2A,C). These results convincingly demonstrate the presence of an omission response in the pupil in both modalities when a stimulus is predicted but unexpectedly omitted. Results also support the notion that the oP3 (presumably belonging to the surprise and orienting-related P3a ERP response family) and omission PDR might reflect at least partly corresponding processes. Indeed, like stimulus deviants, results suggest that surprising omissions can be considered a salient event, with potentially similar effects on behavior like distracted attention (62, 63).

This study is the first to directly compare pupillary omission responses between different modalities. Results show evidence against a difference in omission PDRs between the two modalities. This aligns with the EEG findings of Dercksen et al. (30), who observed similar oP3 somatosensory omission responses to those seen in auditory studies [e.g., (32)]. The similarity in pupil responses between the two modalities suggests similar activation in the brain circuits associated with pupil control, which are thought to include the LC-NE system and the superior colliculus (8, 16–21).

### 4.2. Omission responses in 88%-condition compared to omission responses In 50%-condition

To confirm that the amplified PDR in the 88%-condition was caused by surprise, a 50%-condition included occasional stimuli but, like the motor-control condition, no expectation of stimuli. The cross-condition comparison again involved two identical events (a button press without a stimulus), where now both conditions included stimuli. Contrary to our hypothesis, there was no evidence for a larger omission PDR in the 88%-condition compared to the 50%-condition (Figure 2). This contradicts the results of omission studies using EEG that have consistently reported omission responses when comparing to a 50%-condition (30, 34). Furthermore, the pattern of results was unclear about whether the PDR was different or the same between the 50%-condition and the motor-control condition. This was unexpected as well, as PDRs between these conditions were expected to be similar rather than different given that theoretically omissions in the 50%-condition are assumed to not elicit a surprise response. To address these surprising results, potential confounds were explored by considering block-specific effects, which are known to severely affect pupil responses. For example, a review of Zekveld et al. (22) identified 19 pupillometry studies that explicitly mention habituation effects over the course of experimental blocks. In the current study, the 88%-condition included five blocks, while the 50%-condition included only one block (since more 88%-condition blocks were needed to obtain the same number of omission trials given the different presentation rates). Additionally, the motor-control condition included two blocks (spread out across the experiment). The difference in experimental blocks between conditions carried a risk of disproportionate habituation effects. Post-hoc exploration of the data demonstrated this effect, showing a strong decrease of the omission PDR after the first block of a condition (Figure 3). When only considering the omission trials in the first block of the 88%-condition, the hypothesized larger omission PDR compared with the 50%-condition was observed (Figure 3). The previous effect observed in the 50%-condition compared to the motor-control condition appears to be influenced by block-specific effects as well. When compared with only the first motor-control block, there was evidence against a difference between motor-control and 50%-conditions, in line with the assumption that omissions in the 50%-condition do not elicit surprise. In contrast, a larger omission PDR was still observed when comparing the first block of the 88%-condition with the first block of the motor-control condition. This post-hoc analysis supports the central role of surprise in the omission response and reveals the potential impact of habituation effects. Although habituation effects might be present using other methods than pupillometry (e.g., fMRI, EEG), they are possibly small enough (relative to the omission response) to not interfere with detection of the omission response. Indeed, EEG studies report only small decrements in the P3 elicited by auditory deviants over experimental blocks (64, 65). These results show that future studies, that aim to study omission responses using pupillometry, should aim to balance experimental blocks across conditions in order to increase statistical power and accuracy. If this is not possible, it is important to consider block-specific effects during analysis to avoid erroneous conclusions. Note that the current post-hoc analysis has the limitation that it only controls for block-specific effects within conditions. This confounding effect was expected to be most influential, as participants were instructed before a new condition and likely needed time to get used to it. However, other effects may also have played a role. For example, on average the presentation of the first block of the 88%-condition occurred earlier in the experiment than the 50%-condition.

Previous pupillometry studies typically compared unexpected omissions with standard stimulus responses to assess the presence of an omission response. This approach may be problematic because the pupil dilates in response to both surprise as well as stimuli. Interestingly, the present study demonstrates how this comparison can be influenced by stimulus-related activity and lead to incorrect conclusions regarding the omission response. In the tactile modality, the relatively small PDR to stimuli is similar to the motor-control and thus allows for the detection of the surprise response in the omission PDR (Figure 2C). However, the sound stimulus produced a PDR that completely masked the omission PDR, potentially leading to the erroneous conclusion that no surprise response is present in the pupil (Figure 2A). The different stimulus responses could be attributed to modality-specific factors, but might also be influenced by stimulus characteristics. For example, the sounds used in the current study changed between blocks, were more complex, longer in duration, and arguably more intense than the tactile stimuli, all of which are factors that are known to affect the PDR to stimuli (1, 66–69). Controlling for all stimulus-related factors that affect the PDR can be challenging and conflicts with one of the primary benefits of omission paradigms, which aim to minimize the influence of stimulus-related activity. The potential contribution of stimulus activity to inconsistent or null findings in previous pupillometry studies, particularly in the auditory modality, should be carefully considered.

### 4.3. Applications

This study offers a robust and unconfounded approach for investigating the neural mechanisms of surprise. Understanding how the brain responds to surprise has become increasingly relevant not only for a fundamental understanding of the brain, but also in the context of various clinical disorders. For example, several accounts of autism and schizophrenia put the prediction processes that give rise to surprise (or prediction error) at the core of these disorders (70–72). Given the potential differences in stimulus-related processing between patient and control groups (73), omission studies, such as the one presented in this study, may be particularly well-suited to isolate and study the effect of surprise (as stimulus-related processing is minimized in the omission response). Additionally, pupillometry offers a number of practical advantages that are especially relevant for clinical populations. These include very good signal-to-noise ratio which reduces recording times compared to other methods such as EEG, reduced physical contact during preparation, reduced preparation time, and minimal attributes that need to be attached to the head. Furthermore, the use of remote eye-trackers does not require movement restrictions as is the case using EEG. This is particularly advantageous when investigating prediction and attention processes in patients with hyperactivity (e.g., ADHD) or tremor (e.g., Parkinson’s disease). The combination of a strong indicator of prediction error (the omission response) together with these practical advantages make the current study a useful blueprint for future clinical studies.

## 5. Conclusion

The current study presents conclusive evidence of a pupil response to the unexpected omission of a stimulus, presumably reflecting surprise and the orienting of attention. This finding aligns with the interpretation of the oP3 component observed in EEG omission studies and indicates the involvement of subcortical structures such as the locus coeruleus and superior colliculus in the omission response. There was no indication of an amplitude difference between modalities, suggesting similar, modality-unspecific activation of the involved brain circuits. Additionally, this study was able to identify two important factors that might have contributed to unreliable omission results in past pupillometry studies. First, habituation over experimental blocks apparently plays a substantially larger role in pupillometry compared to EEG studies. Second, stimulus processing effects might mask omission responses when compared with the standard stimulus. These effects should be carefully considered in future study designs. Finally, omission studies using pupillometry may be well suited for use in clinical studies that investigate surprise processing.

## Data availability statement

The raw data supporting the conclusions of this article will be made available by the authors, without undue reservation.

## Ethics statement

The studies involving human participants were reviewed and approved by Ethics committee of the medical faculty of the OVGU Otto von Guericke University, Magdeburg, Germany. The participants provided their written informed consent to participate in this study.

## Author contributions

TD: conceptualization, methodology, software, validation, formal analysis, investigation, data curation, writing—original draft, writing—review and editing, visualization. AW: conceptualization, methodology, software, formal analysis, writing—review and editing. NW: conceptualization, methodology, resources, writing—review and editing, supervision, project administration, funding acquisition. All authors contributed to the article and approved the submitted version.

## Funding

This work was supported by the Center for Behavioral Brain Sciences Magdeburg financed by the European Regional Development Fund (ZS/2016/04/78120) and Leibniz Association (P58/2017). Funding sources did not have any involvement in study design, data collection/analysis/interpretation, writing of the report, or decision to submit for publication.

## Conflict of interest

The authors declare that the research was conducted in the absence of any commercial or financial relationships that could be construed as a potential conflict of interest.

## Publisher’s note

All claims expressed in this article are solely those of the authors and do not necessarily represent those of their affiliated organizations, or those of the publisher, the editors and the reviewers. Any product that may be evaluated in this article, or claim that may be made by its manufacturer, is not guaranteed or endorsed by the publisher.

## Footnotes

^1^Note that the preferable 2 × 2 ANOVA design including the factors expectation (omission vs. motor-control) and modality (auditory vs. somatosensory) would result in an unbalanced design as the motor-control condition was modality unspecific. Therefore, we replaced it by an analogous design including two *t*-tests.
